# Very preterm birth and cognitive control: The mediating roles of motor skills and physical fitness

**DOI:** 10.1016/j.dcn.2021.100956

**Published:** 2021-04-19

**Authors:** Sebastian Ludyga, Uwe Pühse, Markus Gerber, Manuel Mücke, Sakari Lemola, Andrea Capone Mori, Mark Brotzmann, Peter Weber

**Affiliations:** a University of Basel, Department of Sport, Exercise and Health, Basel, Switzerland; b Bielefeld University, Faculty of Psychology and Sports Science, Department of Psychology, Bielefeld, Germany; c Kantonsspital Aarau, Clinic for Children and Teenagers, Division of Neuropediatrics, Aarau, Switzerland; d University of Basel, University Children's Hospital, Division of Neuropediatrics and Developmental Medicine, Basel, Switzerland

**Keywords:** N200, P300, Event-related potentials, Children, Physical fitness

## Abstract

The neurophysiological mechanisms underlying executive function deficits in very preterm born children still remain unclear. Moreover, evidence on factors that can be modified by behavior and exert an influence on these deficits is lacking. The present case-control study examined the association between very preterm birth and neurophysiological indices of response inhibition (i.e. the N200-P300 complex) as well as the potential mediation of this association by aspects of physical fitness. 54 children born very preterm completed a submaximal cycling ergometer test and a motor skill test battery. Event-related potentials elicited by a Go/NoGo task were recorded using electroencephalography. Cases were then matched to full-term children (age: 11 ± 0.7 y). A higher error rate on NoGo trials was found in children born very preterm compared to those born full-term. Path-analyses further revealed that very preterm birth was associated with decreased NoGo P300 amplitude. Motor skills, but not aerobic fitness, fully mediated this association. In early adolescence, very preterm birth is associated with less effective recruitment of attentional resources for stimulus evaluation processes. The improvement of motor skills rather than cardiorespiratory fitness appears promising for reducing this specific impairment in cognitive control.

## Highlights

• Very preterm birth was associated with impaired response inhibition.
• Impaired behavioral performance was linked with decreased P300, but not N200.
• Motor skills fully mediated the association of preterm birth and P300.
• Cardiorespiratory fitness did not contribute to this mediation.
• Improving motor skills might promise the reduction of cognitive control deficits.

## 1 Introduction

About 11 % of all livebirths are born before 37 weeks of gestation and the prevalence increases globally (Blencowe et al., 2012). Whereas there is a trend for reduced mortality rates in preterm neonates (Lui et al., 2019), babies born extremely or very preterm are still facing a wide range of neurodevelopmental disabilities (Cheong et al., 2020; Pascal et al., 2018). With regard to cognitive impairments, meta-analytical findings suggest that preterm children perform 0.5 standardized mean differences below full-term peers on executive function tasks (Van Houdt et al., 2019). Given that the heterogeneity in effect sizes could not be explained by age, deficits in this cognitive domain seem to persist at least throughout childhood. Unfortunately, low executive function has been associated with academic underperformance in early life and lower real-life achievements in later life (Dai et al., 2020; Kroll et al., 2017). Preterm birth further accounts for a three-fold risk for ADHD (Franz et al., 2018), which might partly be attributed to specific impairments in response inhibition (Réveillon et al., 2016). Although this prognosis highlights the need for an early intervention, the neural underpinnings of response inhibition that should to be targeted remain unclear.

Among different neuroimaging methods, event-related potentials (ERPs) recorded via electroencephalography provide an opportunity to study neurocognitive processes. In this respect, a review on auditory ERPs in infants has primarily linked cognitive impairments with late components and mismatch negativity in particular (Depoorter et al., 2018). While these findings indicate early abnormalities in sensory and attentional processes, less is known on the neurophysiological basis of executive function deficits. With regard to response inhibition, the N200 (negative peak within 200−400 ms) and P300 (positive peak within 300−600 ms) allow the examination of processes related to conflict monitoring and the suppression of a prepotent motor response, respectively (Huster et al., 2013; Pires et al., 2014). The amplitudes rather than latencies of these components have been associated with task performance (Barry and Blasio, 2015; Cragg et al., 2009). Both P300 and N200 can be elicited by a Go/NoGo task and are characterized by a fronto-central distribution (Enriquez-Geppert et al., 2010). Whereas the N200 can be detected early during childhood, but decreases in adolescence, the NoGo P3 effect develops around the age of 9 years (Johnstone et al., 2007; Jonkman, 2006). Consequently, preadolescence is an optimal time-window for studying these processes. The few available findings suggest that adolescents born preterm show decreased Go and NoGo P300 along with higher error rate than their full-term peers at 15 years of age (Rommel et al., 2017). Further differences in ERPs were found for the N200 as preterm birth was associated with a decreased amplitude of this component in both trials with low and high inhibitory demands (Rommel et al., 2019). Although these findings indicate that developmental abnormalities in cognitive control processes are still detectable in adolescence, less is known on factors that mediate their association with preterm birth.

Among a variety of factors, aspects of physical fitness need to be considered as potential mediators. Preterm birth is related to persistent deficits in cardiorespiratory fitness (CRF) (Robič Pikel et al., 2017; Smith et al., 2008) and motor skills (Pitcher et al., 2012). These deficits may partly account for impaired cognitive control, given that an interrelation of cognitive and motor development has been suggested (Aadland et al., 2017; Ludyga et al., 2019). Based on a review of the existing literature, this interrelation seems to be pronounced during childhood and preadolescence, but weakens throughout adolescence (van der Fels et al., 2015). A causal relation between physical fitness and cognition is supported by meta-regression findings showing that exercise targeting these aspects elicits small benefits for different cognitive domains, including executive function, across all age groups (Ludyga et al., 2020). Based on the comparison of different training types, coordinative exercise was reported to be most effective, indicating an important role of motor skills for exercise-induced benefits. In children and adolescents, the enhancing effect of exercise on executive function appeared to be stronger for individuals with low compared to high baseline cognitive performance (Ishihara et al., 2020). Exercise interventions might therefore have a high potential to reduce executive function-related deficits in individuals born preterm. However, the development of effective interventions requires knowledge on how changes in physical fitness aspects translate into improvements in cognitive control. In this respect. a recent review found an association of high CRF and high P300 amplitude (Kao et al., 2020). In addition, a lower negativity of N200 was reported in high-fit compared to low-fit children and adolescents (Pontifex et al., 2011; Stroth et al., 2009). A similar pattern of results was obtained for individuals with regular engagement in sports that specifically target motor skills (Li et al., 2019). When interrelations between different physical fitness aspects were accounted for, motor skills were related with increased P300 amplitude, whereas CRF was linked with increased negativity of the N200 amplitude (Ludyga et al., 2021). In summary, the current evidence supports that both physical fitness aspects are related to alterations of the N200-P300 complex. Even though CRF and motor skills may therefore be promising intervention targets, their roles for preterm birth-related deficits in executive function and response inhibition in particular have not yet been examined.

The present case-control study aimed to investigate the association between very preterm birth and behavioral as well as neurophysiological indices of response inhibition (N200, P300) during the transition from childhood to adolescence as well as their mediation by aspects of physical fitness. By accounting for interrelations between motor skills and CRF, their individual contributions to very preterm birth-related response inhibition deficits are examined. Based on the available evidence, we expected an association of very preterm birth with higher error rates and lower P300 and N200 amplitudes (Rommel et al., 2017, 2019). Moreover, we assumed a stronger mediation of these associations by motor skills than CRF (Kao et al., 2020).

## 2 Methods

### 2.1 Participants

Very preterm children (VPC) were recruited from the University Childrens’ Hospital Basel and the Kantonsspital Aarau and matched with full-term peers. Data for full-term control (FTC) cases were collected in a parallel study in the same region, which used identical assessments. Inclusion criteria were 9–13 years of age, right-hand dominance, corrected-to or normal vision as well as very preterm birth for cases (≤32 weeks of gestation) and full-term birth for controls (≥37 weeks of gestation). Common exclusion criteria were any acute or chronic diseases, which were classified as a contraindication for exercise and/ or which impaired the practicability of the scheduled exercise session (e.g. structural birth defects, congenital heart defect, cerebral palsy GMFCS level III–V). Additionally, full-term children with a mental disorder and preterm children with structural epilepsy and/or an intelligence quotient <85 were deemed ineligible. After receiving detailed information on study procedures, written informed consent was obtained from legal guardians of the participants. The study procedures followed the guidelines of the Declaration of Helsinki and were approved by the local ethics committee.

### 2.2 Study design

Participants completed a Go/NoGo task with simultaneous recording of ERPs elicited by the task. To assess socioeconomic status, psychopathology and moderate-to-vigorous physical activity (MVPA), they were asked to fill in the Family Affluence Scale (FAS), the Strengths and Difficulties Questionnaire (SDQ) and the 7-day physical activity recall protocol. Following the administration of the intelligence quotient screening items of the Intelligence and Development Scales-2 (IDS-2) and the collection of anthropometrics, participants’ motor skills and CRF were assessed using the Movement Assessment Battery for children-2 (MABC-2) and the physical work capacity test (PWC170). All measurements were performed during a single laboratory visit, with surrounding noise reduced to a minimum and at an environmental temperature of 21−22 °C.

### 2.3 Motor skills and cardiorespiratory fitness

The MABC-2 consists of age-appropriate fine and gross motor tasks that assess manual dexterity (three items), balance skills (three items) as well as aiming and catching (two items) (Brown, 2018). Standard scores (corrected for age and sex) were derived for each subscale and combined to form a standard score for overall motor skills.

Prior to the PWC170, participants received a heart rate monitor with a chest belt (V800, Polar Electro Oy, Finland). The initial workload was based on the participants’ body weight (<50 kg = 20 W; ≥50 kg = 30 W) and increments depended on the heart rate within the last seconds of each 2-min stage (Bland et al., 2012). When the heart rate reached ≥165 bpm or the participant was not willing to continue, the test was terminated. The workload in the final stage (taking into account the seconds completed in that stage) was divided by the participant’s body weight to calculate relative power output.

### 2.4 Cognitive task

**Fig. A1:**
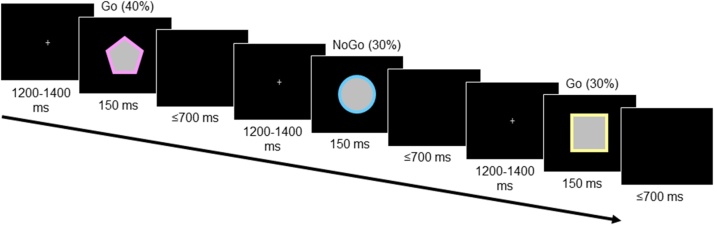
Example trials of the Go/NoGo task.

The Go/NoGo task was administered with E-Prime 3.0 (PST, USA) and included Go trials (30 %), more frequent Go trials (40 %) and NoGo trials (30 %). Participants were instructed to press a button on both Go trial types, but to suppress their motor response on NoGo trials. Speed and accuracy were equally emphasized. Visual stimuli were 10 different two-dimensional, grey geometric shapes (e.g. triangle, square, circle, hexagon) with contour lines drawn either in pink, yellow or blue color (Appendix A, Fig. A1). The colors were matched by intensity and saturation and their occurrence was counter-balanced across the different geometric shapes. Independent of the geometric shape, the color of its contour line indicated the trial type (i.e. more frequent Go trials = pink, Go trials = yellow, NoGo trials = blue) and remained unchanged throughout the task. The presentation of different trial types followed a random order, but was in line with the trial-specific stimulus probabilities. Visual stimuli were displayed against black background for 150 ms, the response window was 850 ms and the inter-trial interval varied randomly between 1200−1400 ms. The task comprised 30 practice trials, followed by three blocks with 100 trials each. Reaction time on response-correct trials, omission (Go and more Frequent Go trials) as well as commission error rates (NoGo trials) were calculated.

### 2.5 EEG recording and processing

The electroencephalogram was recorded from 64 active electrodes (ActiCap, BrainProducts, Germany) arranged according to the 10:10 system (with AFz as ground). Scalp impedance was reduced to 10 KΩ or lower (≥90 % electrodes) by applying a highly conductive gel. Data was referenced to Cz, amplified and digitized at 500 hz (ActiCHamp, BrainProducts, Germany). Offline processing of data was conducted with BESA Research 7.0 (Brain Electric Source Analysis, Germany). Adaptive artefact correction was performed on blinks using spatial filtering based on artefact and brain activity topographies (Ille et al., 2002). In short, this approach separates brain and artefact activities by employing principal component analysis (components explaining more than 1 % variance are maintained). Using all topographies, the recorded data is then decomposed into a linear model containing both brain and artefact activities, so that the estimated artefact signals can be removed with minimal distortion. Artifacts that survived the correction procedure were rejected using individual amplitude (120 ± 22 μV) and gradient (75 ± 2 μV) thresholds. In the next step, recorded data was submitted to low- (zero-phase shift of 30 Hz; slope 24 dB/octave) and high-pass filtering (forward phase shift of 0.1 Hz; slope 6 dB/octave) as well as baseline correction (−200 ms to stimulus onset). On average, 63.3 ± 12.2 Go and 46.5 ± 13.3 NoGo trials with correct responses survived artifact correction and rejection. Grand averaged segments were created for these trial types. In contrast, more frequent Go trials were disregarded to avoid a reduction of the comparability of ERP waves due to different segment numbers used for creating the grand average. Following re-referencing to average mastoids, the fronto-central mean amplitudes in the latency ranges from 250 to 350 ms (Fz. F1, F2, FCz, FC1, FC2) and 400 to 600 ms (FCz, FC1, FC2, Cz, C1, C2) were used to derive the N200 and P300 (Huster et al., 2013), respectively.

### 2.6 Statistical analyses

Statistical analyses were performed using SPSS 25.0 and AMOS 25.0 (IBM, Armonk, USA). The FUZZY plugin was employed for case-control matching based on sex and age. Following the verification of the Gaussian distribution of data with the Shapiro Wilk test, one-way ANOVAs were applied to compare participants’ characteristics (gestational age, anthropometrics, MVPA, psychopathology, intelligence, socio-economic status) and physical fitness (MABC-2, PWC170) between VPC and FTC. To guide the selection of potential confounders, zero-order correlations examined the relation between participants’ characteristics, physical fitness and behavioral as well as ERP outcomes. Participants’ characteristics showing a statistically significant and/or at least moderate correlation with one or more mediators and/or outcomes were controlled for in subsequent path-analyses. Using pre-specified models, the relations of very preterm birth (coded 0 = FTC, 1 = VPC) with behavioral performance (model 1a) and event-related potentials elicited from NoGo (model 2a) and Go trials (model 3a) were examined. In the next step, MABC-2 score and PWC170 were introduced to investigate whether these aspects of fitness mediated the relation of very preterm birth with behavioral performance (model 1b) and event-related potentials elicited from NoGo (model 2b) and Go trials (model 3b). Interrelations among mediators, behavioral and ERP outcomes were accounted for by estimating their covariances. Additionally, participants’ characteristics with a potential confounding effect (as identified in zero-order correlations) were also controlled by covariance estimation. Standardized regression coefficients were calculated to estimate the relative strengths of the examined associations. Furthermore, the hypothesis that coefficients in path-analyses equal zero was examined with *t*-Tests and rejected at *p* < 0.05. Model fit was tested for all models and considered good at RMSEA≤0.08 and χ^2^/df≤2 (Iacobucci, 2010).

## 3 Results

### 3.1 Participants’ characteristics

**Fig. 1:**
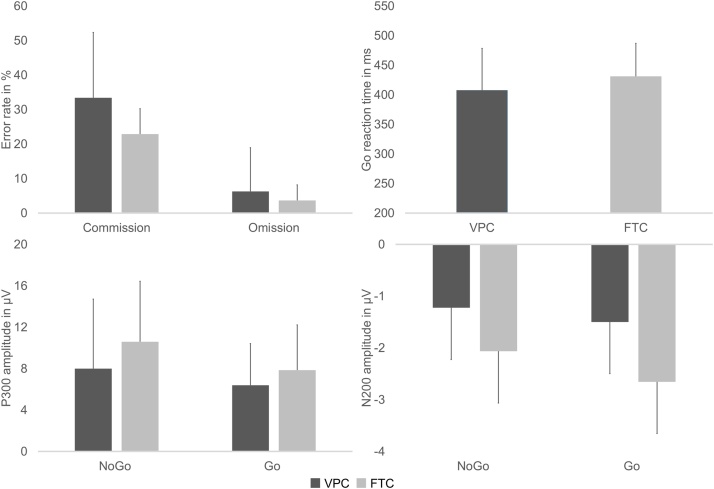
Behavioral performance on the Go/NoGo task and amplitudes of event-related potential components (means and standard deviation) displayed for children born very preterm (VPC) and full-term peers (FTC).

Fifty-four VPC were recruited, completed all assessments and were matched to 54 FTC (from a pool of 95 participants). Whereas no group differences were found for age, weight, body mass index, MVPA, PWC170, IDS-2 and FAS scores, VPC showed a higher SDQ score, *F*(1,105) = 4.21, η² = 0.040, *p* = 0.040, and lower MABC-2 score than FTC, *F*(1,105) = 4.21, η² = 0.040, *p* = 0.040 (Table 1). With regard to potential confounding effects of participants’ characteristics, zero-order correlations revealed statistically significant and/or moderate correlations between body mass index, IDS-2 score, SDQ score, moderate-to-vigorous physical activity and at least one mediator or outcome (Appendix A, Table A1). Behavioral performance on the Go/NoGo task and amplitudes of event-related potential components elicited by the task are shown in Fig. 1.

**Table 1:** Comparison of anthropometrics, physical fitness, intelligence, socioeconomic status, and physical activity between participants born very preterm (VPC) and those born full-term (FTC).

	VPC (N = 29 f/25 m)		FTC (N = 29 f/25 m)		
	*M*	*SD*	*M*	*SD*	*F*
Gestational age in w	29.7	2.1	39.1	1.5	742.42*
Age in y	11.0	1.8	11.0	1.6	0.00
Weight in kg	37.0	9.4	39.6	10.9	1.87
BMI in kg· m^−2^	17.3	2.5	17.6	2.6	0.49
IDS-2 screening score	5.2	3.3	5.9	2.9	1.33
SDQ score	12.1	5.0	10.3	4.0	4.21*
FAS score	6.7	1.6	6.5	1.8	0.46
MVPA in min·d^−1^	49.5	59.9	49.9	31.5	0.00
MABC-2 score	45.9	28.0	64.4	22.2	10.82*
PWC170 in W· kg^−1^	2.0	0.5	2.2	0.5	3.63

BMI = Body mass index; IDS-2=Intelligence and Development Scales-2 (value points of short screening); MABC-2=Motor Assessment Battery for Children-2; MVPA = Moderate-to-vigorous physical activity; PWC170=Physical work capacity at 170 bpm; SDQ = Strengths and Difficulties Questionnaire.

* p < 0.05 full-term vs preterm born participants.

### 3.2 Behavioral performance

**Fig. 2:**
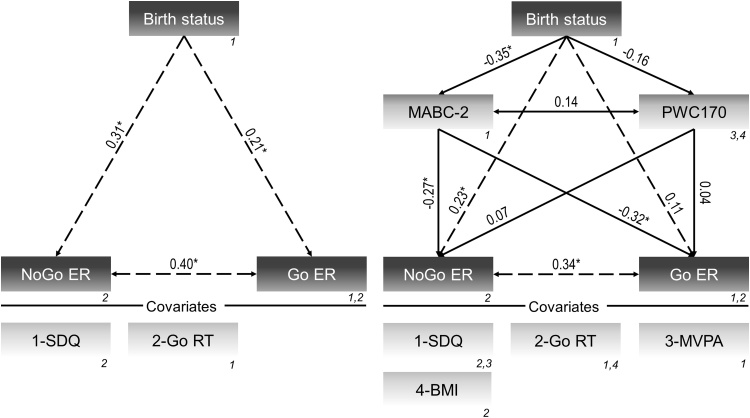
Models investigating the relation of birth status (0=full-term; 1=very preterm) with behavioral performance on the Go/NoGo task (left panel) and its mediation by motor skills and cardiorespiratory fitness (right panel). *Notes:* Numbers in italics show the covariances that were estimated. All covariates included in the models are shown below the vertical line. *p < 0.05; BMI=Body mass index; Go RT=Reaction time on Go trials; MABC-2=Movement Assessment Battery for Children-2; MVPA=Moderate-to-vigorous physical activity; PWC170=Relative physical working capacity at 170 bpm; SDQ=Strengths and Difficulties Questionnaire sum score.

The initial model without mediators (model 1a) showed that very preterm birth was associated with higher commission, β = 0.31, *p* < 0.001, and omission error rates, β=0.21, *p* = 0.024. Following the inclusion of MABC-2 score and PWC170 (model 1b), there was a decrease of regression coefficients from moderate to small magnitude for commission error rate and small to negligible magnitude for omission error rate (Fig. 2). This reduction was explained by an inverse moderate relation of very preterm birth with MABC-2 score, β=-0.35, *p* < 0.001, which in turn was associated with decreased commission, β=-0.27, *p* = 0.001, and omission error rates, β=-0.32, *p* < 0.001. Good model fits was given for both the initial and the meditation models, RMSEA≤0.08, χ^2^/df≤1.65.

### 3.3 Event-related potentials

**Fig. 3:**
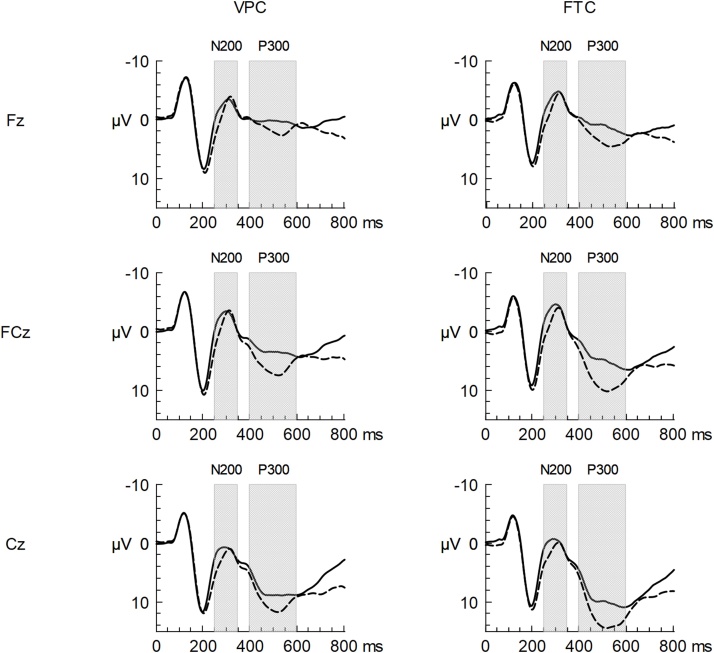
Event-related potential waveforms at fronto-central region displayed for participants born very preterm and those born full-term. *Notes:* Solid lines represent the Go condition, whereas dashed lines represent the NoGo condition.

**Fig. 4:**
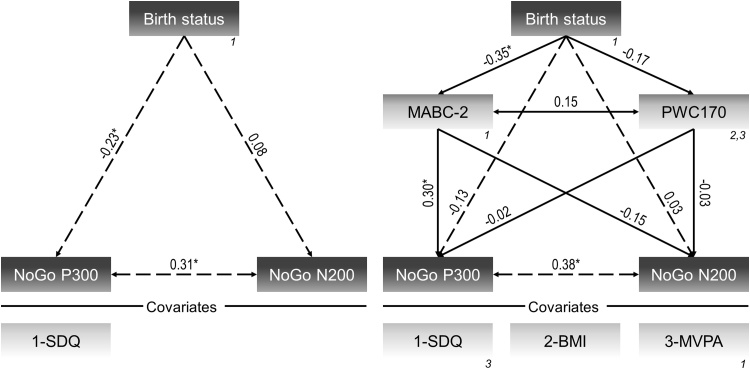
Models investigating the relation of birth status (0=full-term; 1=very preterm) with event-related potential components elicited by NoGo trials (left panel) and its mediation by motor skills and cardiorespiratory fitness (right panel). *Notes:* Numbers in italics show the covariances that were estimated. All covariates included in the models are shown below the vertical line. *p < 0.05; BMI=Body mass index; MABC-2=Movement Assessment Battery for Children-2; MVPA=Moderate-to-vigorous physical activity; PWC170=Relative physical working capacity at 170 bpm; SDQ=Strengths and Difficulties Questionnaire sum score.

Event-related potential waveforms of FTC and VPC are displayed in Fig. 3. Path-analysis showed that very preterm birth was associated with lower NoGo P300 amplitude (model 2a), β=-0.23, *p* = 0.014. When MABC-2 score and PWC170 were included to investigate mediating effects (model 2b), this association decreased and did no longer reach statistical significance (Fig. 4). This reduction was due to the inverse moderate relation of very preterm birth with MABC-2, β=-0.35, *p* < 0.001, and MABC-2 with NoGo P300 amplitude, β=0.30, *p* = 0.002.

**Fig. 5:**
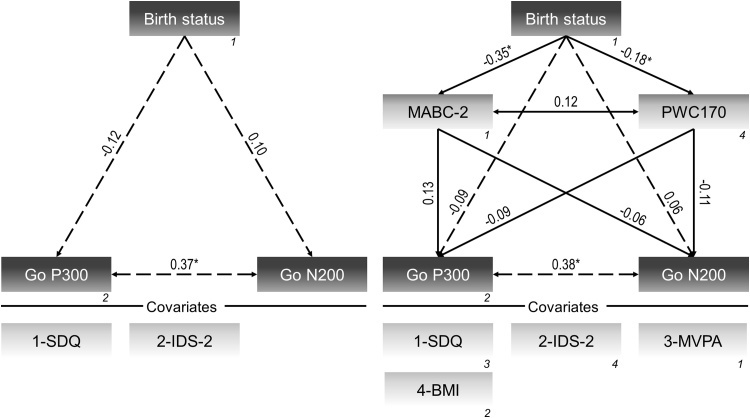
Models investigating the relation of birth status (0=full-term; 1=very preterm) with event-related potential components elicited by Go trials (left panel) and its mediation by motor skills and cardiorespiratory fitness (right panel). *Notes:* Numbers in italics show the covariances that were estimated. All covariates included in the models are shown below the vertical line. *p < 0.05; BMI=Body mass index; IDS-2=Intelligence and Development Scales-2 (value points of short screening); MABC-2=Movement Assessment Battery for Children-2; MVPA=Moderate-to-vigorous physical activity; PWC170=Relative physical working capacity at 170 bpm; SDQ=Strengths and Difficulties Questionnaire sum score.

With regard to the Go condition, there was no statistically significant relation between birth status and both P300 and N200 amplitudes (model 3a). After including the mediators (model 3b), these associations remained unchanged (Fig. 5). Pre-specified models testing the direct and indirect effects showed good model fit, regardless of the assessed condition of the Go/NoGo task, RMSEA ≤ 0.01, χ^2^/df≤0.96.

## 4 Discussion

The investigation of behavioral performance revealed that very preterm birth was associated with high commission and omission error rates, even when reaction time was accounted for. On a neurocognitive level, very preterm birth was further associated with a decreased P300 amplitude in the NoGo condition. When indirect effects via aspects of physical fitness were tested, motor skills mediated the relation of very preterm birth with both behavioral performance and the NoGo P300 amplitude.

The present findings on the N200-P300 complex broaden the understanding of very preterm birth-related developmental abnormalities in response inhibition processes, which seem to persist at least into preadolescence. Whereas conflict monitoring indexed by the N200 amplitude was not associated with birth status, a lower P300 amplitude elicited from NoGo trials was found in VPC compared to FTC. This pattern of results is consistent with a previous study that examined ERPs elicited by a similar cognitive task (Rommel et al., 2017). The NoGo P300 is suggested to reflect frontal lobe engagement in connection with evaluation and updating processes in response to the NoGo stimulus (Huster et al., 2013; Pires et al., 2014). Consequently, VPC appear to have difficulties in the engagement of focal attention for the evaluation of inhibitory processes. Given the moderate inverse correlation between P300 amplitude and commission error rate (Johnstone et al., 2007; Jonkman, 2006), these difficulties may partly account for the very preterm birth-related deficits in behavioral performance.

With regard to physical fitness, VPC showed lower proficiency in motor skills and a tendency towards lower CRF than FTC. However, only motor skills mediated the association between very preterm birth, behavioral performance and the NoGo P300 amplitude. This is a first indication that the improvement of motor skills may benefit response inhibition by more effective recruitment of attentional resources for stimulus evaluation. In this respect, motor skills have previously been linked with the inhibitory aspect of executive function (Aadland et al., 2017; Ludyga et al., 2019; van der Fels et al., 2019). The engagement in similar processes might account for this, since maintaining goal-relevant information, focusing attention, and evaluating visual stimuli to guide a motor response or its suppression are required for both the MABC-2 items and the Go/NoGo task. Electrophysiological evidence partly supports this idea of shared abilities, since the P300 component elicited by NoGo trials can also be observed in motor planning and learning tasks. A review on this topic has attributed the P300 to conscious encoding of target locations (in reaching tasks) and/ or movement amplitude for initiating a subsequent movement (Krigolson et al., 2015). Consequently, the achievement of high motor skills in VPC may be linked with improved feed-forward mechanisms, which can be employed for the subsequent suppression of a motor response in NoGo trials. The explanation of the mediating role of motor skills by shared cognitive processes can further be extended to a common neural substrate. Very preterm birth has been related to grey and white matter alterations in several brain structures, including the inferior frontal gyrus (Nosarti et al., 2014). By means of source localization, this region was found to be the main neural generator of the NoGo P300 (Enriquez-Geppert et al., 2010; Huster et al., 2013). Interestingly, meta-analytical findings revealed that cognitive and motor skill training both lead to a decrease of activity in neural networks associated with cognitive control processes, which also encompasses the inferior frontal gyrus (Lohse et al., 2014). This has been interpreted as increased efficiency of these networks with increasing level of expertise. Therefore, a possible interpretation of the mediating role of motor skills is that the acquisition of such skills might allow for a compensation of abnormalities in specific brain structures by promoting their efficiency.

In contrast to motor skills, CRF did not mediate the association between very preterm birth and decreased NoGo P300 amplitude. Based on the inspection of regression paths, mainly the absence of a correlation between PWC170 and P300 amplitude accounted for this. It should be noted that this finding is in conflict with a previous review reporting strong evidence for a higher P300 amplitude in individuals with high CRF (Kao et al., 2020). However, the majority of the reviewed studies that investigated this ERP component from an inhibitory control task employed a paradigm demanding interference (*k* = 26) rather than response inhibition (*k* = 2). Thus, a moderation of the association between CRF and inhibitory control by its subtype cannot be ruled out. In the context of the present study, this implies that the promotion of motor skills rather than CRF has potential for counteracting the impaired cognitive control process indexed by reduced P300 amplitude in VPC.

Although the present findings provide first indications on the aspect of fitness that should be targeted to improve response inhibition, they should be interpreted with caution due to the cross-sectional design. Moreover, it remains unclear whether the mediating role of motor skills can be generalized to other components of executive function. Similarly, no conclusions can be drawn on the role of gestational age among preterm children, meaning that those born late preterm may lack a decreased NoGo P300 amplitude. The same applies to a potential influence of the age at assessment. Thus, the present findings provide no indication on whether the observed NoGo P300 abnormalities persist or are no longer detectable in later stages of life. With regard to the methodology, the current study was limited to the examination of the N200-P300 complex due to the chosen Go/NoGo paradigm, so that it remains unclear if VPC also show abnormalities in other processes related to response inhibition. Furthermore, despite the use of case-control matching and the consideration of many confounders, not all potential sources of variance in the mediators and outcomes could be accounted for (e.g. pubertal status). Additionally, only motor skills and CRF were assessed, which allows no conclusions on the role of other aspects of physical fitness for cognitive control processes in VPC.

## 5 Conclusions

In preadolescence, children born very preterm show impaired response inhibition in comparison to their full-term peers. On a neurocognitive level, this becomes evident by a reduced engagement of focal attention for evaluation processes that guide the subsequent selection of an appropriate motor response or its suppression. The associations of very preterm birth with both behavioral and neurocognitive impairments in cognitive control are mediated by motor skills. This provides a first indication that interventions should target such skills to reduce preterm birth-related deficits in response inhibition.

## Data statement

Data will be made available on request to the corresponding author.

## Funding

The collection of data on very preterm children was funded by the Forschungsfonds of the University of Basel. The collection of data on full-term children was funded by the OPO Foundation and the Freiwillige Akademische Gesellschaft Basel.

## Declaration of Competing Interest

The authors report no declarations of interest.

## Figures and Tables

**Table A1:** Correlations between potential confounders, mediators and outcomes.

	BMI	IDS-2	SDQ	FAS	MVPA
MABC-2	−0.14	0.08	−0.30*	0.02	0.10
PWC170	−0.39*	−0.01	−0.06	0.17	0.28*
Go reaction time	−0.33*	0.29*	0.22*	−0.08	−0.07
Omission error rate	0.15	0.08	0.20*	−0.15	−0.16
Commission error rate	0.16	−0.04	0.09	−0.03	−0.09
Go N200	0.04	0.10	0.05	−0.07	−0.06
Go P300	−0.13	0.23*	0.03	0.00	−0.15
NoGo N200	0.07	0.03	0.00	−0.04	−0.15
NoGo P300	−0.06	0.05	−0.10	0.08	−0.01

BMI=Body mass index; FAS=Family Affluence Scale; IDS-2=Intelligence and Development Scales-2 (value points of short screening); MABC-2=Movement Assessment Battery for Children-2; MVPA=Moderate-to-vigorous physical activity; PWC170=Relative physical working capacity at 170 bpm; SDQ=Strengths and Difficulties Questionnaire sum score.

* *p* < 0.05.
